# Development of a simple intensified fermentation strategy for growth of *Magnetospirillum gryphiswaldense* MSR-1: Physiological responses to changing environmental conditions

**DOI:** 10.1016/j.nbt.2018.05.1201

**Published:** 2018-11-25

**Authors:** Alfred Fernández-Castané, Hong Li, Owen R.T. Thomas, Tim W. Overton

**Affiliations:** a School of Chemical Engineering, University of Birmingham, B15 2TT, Birmingham, UK; b Institute of Microbiology & Infection, University of Birmingham, B15 2TT, Birmingham, UK

**Keywords:** BOX, Bis-(1,3-Dibutylbarbituric Acid Trimethine Oxonol, DCW, dry cell weight, FCM, flow cytometry, FSM, flask standard medium, MTB, magnetotactic bacteria, PHA, polyhydroxyalkanoate, PI, propidium Iodide, Pyr546, Pyrromethene-546, Magnetosomes, Flow cytometry, Physiology of magnetotactic bacteria, pH-stat fermentation

## Abstract

The development of a simple pH-stat fed-batch fermentation strategy for the production of *Magnetospirillum gryphiswaldense* MSR-1 and magnetosomes (nanoscale magnetic organelles with biotechnological applications) is described. Flow cytometry was exploited as a powerful analytical tool for process development, enabling rapid monitoring of cell morphology, physiology and polyhydroxyalkanoate production. The pH-stat fed-batch growth strategy was developed by varying the concentrations of the carbon source (lactic acid) and the alternative electron acceptor (sodium nitrate) in the feed. Growth conditions were optimized on the basis of biomass concentration, cellular magnetism (indicative of magnetosome production), and intracellular iron concentration. The highest biomass concentration and cellular iron content achieved were an optical density at 565 nm of 15.5 (equivalent to 4.2 g DCW·L^−1^) and 33.1 mg iron·g^−1^ DCW, respectively. This study demonstrates the importance of analyzing bacterial physiology during fermentation development and will potentially aid the industrial production of magnetosomes, which can be used in a wide range of biotechnology and healthcare applications.

## Highlights

• Magnetosomes are natural intracellular, membrane-bound, magnetic nanoparticles.
• Magnetosomes have a variety of clinical and biotechnological applications.
• Magnetosomes are currently difficult to produce at large scale.
• We developed a simple, scalable, fermentation strategy for magnetosome production.
• The methods developed will aid development of magnetosome technologies.

## Introduction

Magnetotactic bacteria (MTB) are a phylogenetically diverse group of bacteria that are able to synthesize magnetosomes; sub-cellular nanoscale organelles that comprise chains of crystals of magnetite or greigite (depending upon the species of MTB), with each crystal coated in a biological phospholipid membrane containing membrane proteins [1,2]. Magnetosomes represent an attractive alternative to existing commercially available chemically-synthesized magnetic nanoparticles, the synthesis of which usually requires: extreme temperature conditions (∼320 °C in the “heat up” method); organic solvents; and subsequent complex in vitro surface modification steps for grafting biomolecules to the particle surface [3]. Chemical synthesis of magnetic nanoparticles with a narrow size range and at large scale is also difficult [[4], [5], [6]]. Magnetosomes therefore have several advantageous properties: they are ferrimagnetic; have a narrow size distribution; are coated in organic material, preventing aggregation [1,2]; and can be functionalized in vivo using genetic engineering tools, allowing one-step manufacture of functionalized particles [7].

Magnetosomes have been utilized in a wide range of biotechnological and healthcare applications, such as: contrast agents for magnetic resonance imaging; development of immunoassays; cell sorting and cancer therapy [[8], [9], [10], [11]]. However, availability of magnetosomes in sufficient quantities for these applications is problematic due to the low magnetosome yield of naturally occurring MTB species. *Magnetospirillum gryphiswaldense* is an MTB that has been subject of considerable research and can be cultured at higher cell densities than other MTB species; strain MSR-1 also generates up to 4% of its dry cell weight as magnetosomes [12]. Recent studies have optimized fermentations and employed genetic engineering approaches to improve magnetosome yields. Nevertheless, further improvement of MTB biomass and magnetosome yields remains a key objective in the field.

The production of magnetosomes in *M. gryphiswaldense* occurs under oxygen-limited conditions; magnetite biomineralization is induced only below a threshold value of 20 mbar O_2_ and optimum conditions for magnetosome formation were found at pO_2_ = 0.25 mbar [2]. In order to achieve such conditions, sophisticated control regimes employed gas blenders to supply mixtures of nitrogen and air containing 1% O_2_ to maintain microaerobic conditions [2]. This strategy requires supplying relatively high gas flow rates (0.1–3 L·min^−1^), leading to potential foaming in bioreactors and consequent use of antifoams, which may impair growth [13]. The complexity of gas blending and expense of very sensitive pO_2_ probes renders scaleup difficult and unattractive. In other studies, the fermenter was supplied with air and the pO_2_ was maintained between 0 and 1% by regulating airflow and agitation through cascade control [14]; alternatively, highly sensitive pO_2_ probes were employed for accurate monitoring of absolute values of O_2_ in the parts per billion range [15]. However, using cascade control to maintain pO_2_ set-point is likely to cause oscillations in dissolved oxygen concentration resulting in unstable pO_2_ in the fermenter [16]. Therefore, design of methods for growth of MTB in bioreactors must pay particular attention to the stable control of dissolved oxygen concentration. Efforts should also be made to employ control that can be scaled up for eventual industrial production of MTB.

In addition to oxygen concentration, denitrification pathways have been shown to be important for magnetosome formation [[17], [18], [19]]. Magnetite biomineralization has been linked to dissimilatory nitrate reduction to dinitrogen gas, employing a periplasmic Nap nitrate reductase, cytochrome *cd*_1_-dependent NirS nitrite reductase, a NorBC nitric oxide reductase and NosZ N_2_O reductase [17,18]. The highest expression of denitrification-related genes coincided with conditions permitting maximum magnetite synthesis [17]. Both oxygen and nitrate are used as electron acceptors under microaerobic conditions, the former being reduced by a high affinity *cbb*_3_-type terminal oxidase [19]. A homologue of the oxygen-sensing transcription regulator FNR, MgFnr, regulates these processes and thus is important for magnetosome synthesis [19].

The physiology of *M. gryphiswaldense* has not been sufficiently studied in high-cell density cultures and so little is known about the parameters that limit biomass and magnetosome yields. For other organisms, yields are determined by factors such as media formulation, feeding strategy, bioreactor mixing and/or oxygen availability. Typically, for *M. gryphiswaldense*, shake flask cultures with limited control of process parameters yield biomass equivalent to an OD_565_ of 1–2 [2,20] and therefore, findings from such shake flask cultures are not transferrable to bioreactor cultures, where biomass concentrations are around 10-fold higher. So far, *M. gryphiswaldense* MSR-1 has been the most studied strain in bioreactor experiments [21,22]. Zhang and co-workers [14] examined the increase of osmotic potential as a function of media composition. Yang et al. [15] investigated physiological and metabolic stress parameters, such as reducing power and ATP content, to reveal conditions for magnetosome formation. More recently, transcriptome analysis was used to compare magnetosome forming and non-forming conditions in *M. gryphiswaldense* in fermentation experiments [23].

In this study, we describe the development of a simple pH-stat based fermentation strategy that affords growth of *M. gryphiswaldense* MSR-1 to high cell densities. We have employed flow cytometry methods recently developed in our laboratory [24] to evaluate a range of physiological and stress parameters (cell morphology, aspects of metabolism, and the accumulation of intracellular polyhydroxyalkanoate (PHA)), and investigated the impact of different concentrations of lactate and nitrate on cell growth and magnetosome formation. We aim to better understand the bacterial physiology and metabolism of MTB, so that they might be grown industrially at large scale to high cell densities with elevated magnetosome content, allowing development of magnetosome-related applications [2,14,25].

## Materials and methods

### Strains, growth media and culture conditions

*Magnetospirillum gryphiswaldense* MSR-1 was obtained from Deutsche Sammlung von Mikroorganismen und Zellkulturen GmbH (DSMZ, Germany) and used for all experiments. Unless indicated otherwise, all chemicals were purchased from Sigma-Aldrich (Poole, UK). Cryostocks of *M. gryphiswaldense* in 5% DMSO were routinely grown in flask standard medium (FSM) comprising: 3.5 g·L^−1^ potassium l-lactate; 100 μM iron citrate (C_6_H_5_FeO_7_); 0.1 g·L^−1^ KH_2_PO_4_; 0.15 g·L^−1^ MgSO_4_·7H_2_O; 2.38 g·L^−1^ HEPES; 0.34 g·L^−1^ NaNO_3_; 0.1 g·L^−1^ yeast extract; 3 g·L^−1^ soy bean peptone; and 5 mL·L^−1^ EDTA-chelated trace element solution (EDTA-TES [26]) replacing MnCl_2_ for MnSO_4_·H_2_O. EDTA-TES contained: 5.2 g·L^−1^ EDTA disodium salt; 2.1 g·L^−1^ FeSO_4_·7H_2_O; 30 mg·L^−1^ H_3_BO_3_; 85.4 mg·L^−1^ MnSO_4_·H_2_O; 190 mg·L^−1^ CoCl_2_ g·L^−1^; 4 mg·L^−1^ NiCl_2_·6H_2_O; 2 mg·L^−1^ CuCl_2_·2H_2_O; 44 mg·L^−1^ ZnSO_4_·7H_2_O and 36 mg·L^−1^ Na_2_MoO_4_·2H_2_O. Pre-cultures used for bioreactor inoculation were grown in FSM without iron source. The pH of FSM was adjusted to 7.0 with NaOH [2]. Cells were grown at 30 °C in flat-bottomed flasks in an orbital shaker incubator operated at 150 rpm.

The batch medium for bioreactor experiments consisted of FSM without iron citrate and the feed solution contained: 50–200 g·L^−1^ lactic acid; 3–25 g·L^−1^ NaNO_3_; 18 mL·L^−1^ 25–28% NH_3_·H_2_O; 6 g·L^−1^ yeast extract; 2.4 g·L^−1^ MgSO_4_·7H_2_O; 6 g·L^−1^ K_2_HPO_4_·3H_2_O; 70 mL·L^−1^ Mineral Elixir and 2 g·L^−1^ FeCl_3_·6H_2_O. The mineral elixir (pH 7) contained: 1.5 g·L^−1^ nitrilotriacetic acid; 3 g·L^−1^ MgSO_4_·7H_2_O; 0.5 g·L^−1^ MnSO_4_·2H_2_O; 1 g·L^−1^ NaCl; 0.1 g·L^−1^ FeSO_4_·7H_2_O; 0.18 g·L^−1^ CoSO_4_·7H_2_O; 0.1 g·L^−1^ CaCl_2_·2H_2_O; 0.18 g·L^−1^ ZnSO_4_·7H_2_O; 0.01 g·L^−1^ CuSO_4_·5H_2_O; 0.02 g·L^−1^ KAl(SO_4_)_2_·12H_2_O; 0.01 g·L^−1^ H_3_BO_3_; 0.01 g·L^−1^ Na_2_MoO_4_·2H_2_O; 0.03 g·L^−1^ NiCl_2_·6H_2_O and 0.3 mg·L^−1^ Na_2_SeO_3_·5H_2_O.

### Bioreactor set up

An Electrolab (Tewkesbury, UK) Fermac 310/60 5-L jar bioreactor equipped with 4 baffles and an agitator with 2 six-bladed Rushton turbines was used. Aeration was achieved by sparging air from below the lower impeller at a rate of 0 – 100 mL·min^−1^, through a reusable, autoclavable 0.22-μm filter (Sartorius). Dissolved oxygen in the medium (pO_2_) was measured online using a D150 Oxyprobe (Broadley James). Agitation was maintained at 100–500 rpm. pH was measured using an F-695 FermProbe (Broadley James) and was controlled at a set-point of 7 ± 0.05 with the automated addition of an acidic feeding solution. Off-gas passed through a condenser, autoclavable 0.22-μm filter (Sartorius, Goettingen, Germany) and HEPA filter (Millipore, Darmstadt, Germany). The temperature was maintained at 30 °C by a heating jacket and coldfinger. Routine operational conditions were developed in this study and are detailed in the Results and Discussion section.

### Flow cytometry

Bacteria were analyzed using a BD Accuri C6 flow cytometer (BD Biosciences, UK). Samples were taken from the bioreactor, resuspended in phosphate-buffered saline (PBS) and stained with dyes listed in Table S1. Samples were excited with a 488 nm and forward scatter (FSC) and side scatter (SSC) and fluorescence data collected (Table S1). Dyes were used for fluorescence assays and with the exception of Pyr546 (Photonic solutions, Ohio, USA) were purchased from Thermo Fisher Scientific (UK).

### Analytical methods

Culture optical densities at 565 nm (OD_565_) were measured using a spectrophotometer (Evolution 300 UV–vis, Thermo Scientific, UK). Data were collected using VISIONpro software. Magnetic response (C_mag_) of cells was measured immediately after OD_565_ using a magnetic measurement system built into the spectrophotometer, based on devices described in the literature [27,28]. Briefly, 2 pairs of Helmholtz coils were arranged around the cuvette holder, one pair perpendicular to the light beam and the other pair in line with the light beam. Each pair of coils was energized in turn (producing a magnetic flux density of 1.9 m T at the center of the cuvette) and the optical density (OD_565_) measured in each state. Magnetic cells align with the magnetic field and thus are either oriented in line with the light beam, or perpendicular to it, thereby changing the optical density measurement. Non-magnetic cells do not move when the magnetic field is changed, so their optical density is identical in both states. The C_mag_ is calculated by dividing the OD_565_ value measured when cells are aligned parallel to the light beam by that obtained when cells are aligned perpendicular to the light beam. For dry cell weight (DCW) determination, 1 mL samples, prepared in triplicate, were centrifuged and washed three times with MilliQ water, followed by overnight incubation at 105 °C.

Fluorescence microscopy (Zeiss Axiolab) was used to observe stained cells with fluorescent probes. Images were acquired using an AxioCam ICm1 camera and processed using ZEN Lite 2012 software in auto exposure mode. Samples were excited using an ultraviolet light source (Zeiss VHW 50f-2b) and fluorescence was detected using a 520 L P filter.

### Nitrate and nitrite assays

Nitrate concentration was determined using the Szechrome NAS reagent system (Polysciences inc., USA) according to manufacturer’s instructions. Briefly, a working reagent was prepared comprising 5 g·L^−1^ Szechrome NAS reagent dissolved in a 1:1 mixture of 85–86% (v/v) H_3_PO_4_ and 95–97% H_2_SO_4_. Samples were diluted 40-fold before analysis, prepared in duplicate, and 50 μL portions were pipetted into 1 cm light path cuvettes, before adding 950 μL of working reagent and incubating for 1 h. Absorbance was read at 570 nm in a spectrophotometer (Evolution 300, Thermo Scientific, USA).

Nitrite concentration was determined using the Greiss reagent kit system (Promega, USA) according to manufacturer’s instructions. Samples were diluted 20-fold for analysis, prepared in duplicate, and absorbance was read at 560 nm in a plate reader.

### Lactic acid assay

Extracellular l-lactic acid concentration was measured using an l-lactic acid kit (Megazyme, Ireland) according to manufacturer’s instructions. Samples were diluted 40-fold for analysis in duplicate, and reactions were prepared at total volumes of 1 mL in 1 cm light path cuvettes. Absorbance was measured at 340 nm before and after an incubation time of 10 min.

### Iron concentration

Flame atomic absorption spectroscopy was performed as an offline analysis to study the intracellular and extracellular iron concentrations within the bioreactor. Iron was determined at 248.3 nm using a single element iron hollow cathode lamp (SMI-LabHut Ltd.) operated at a current of 30 mA with an acetylene/air flame (0.7 L·min^−1^ acetylene and 4.0 L·min^−1^ air) in a Perkin Elmer AAnalyst 300 Atomic Absorption Spectrometer (USA). Sample preparation was done in triplicates as described elsewhere [2]. Briefly, 500 μL nitric acid (70% v/v) was used to solubilize the iron in the form of magnetite pellets and incubated at 98 °C for 2 h with shaking at 300 rpm, whereas 10 μL were employed for supernatant samples.

### Transmission electron microscopy

Cell pellets were centrifuged at 14,000 rpm for 3 min, resuspended in 1 mL of 2.5% (v/v) glutaraldehyde in 0.1 M potassium phosphate solution (pH 7.2) and incubated for 1 h. A series of washing steps with increasing alcohol concentration (50–100% v/v) followed. Sedimented cells from the last dehydration step were embedded in resin by infiltration of the pellet with a solution containing 50% (v/v) Mollenhauer [29] resin in propylene oxide (Agar Scientific) on a rotator (Type N, TAAB) operated at 4 rpm for 12 h in a fume cupboard, followed by curing in undiluted Mollenhauer resin at 60 °C for another 48 h. Thin sections (120 nm) were cut from the resin block using diamond knives on a Reichert-Jung UltraCut Ultramicrotome. The cut sections were examined using a JEOL 1200EX TEM electron microscope operated at 80 keV, in the transmission mode, with the beam current at 60 μA.

## Results and discussion

This study investigated the production of magnetosomes in *M. gryphiswaldense* MSR-1 in bioreactors upon variation of environmental conditions and feed composition (lactic acid and sodium nitrate concentration). Magnetosome biomineralization has been shown to occur under microaerobic conditions [2] and previous studies performed in bioreactors demonstrated that optimal process conditions to obtain high biomass and magnetosome yields were achieved by balancing O_2_ and nutrient supply [2,14]. Here, a relatively simple intensified fermentation strategy was developed using flow cytometry (FCM) to study changes in cell physiology and morphology, PHA accumulation, and intracellular chelatable iron concentration. First, scoping fermentations were carried out to define a routine fed-batch pH-stat growth strategy. The pH-stat strategy was first used by Zhang et al. [14] for *M. gryphiswaldense* growth; an acidic feeding solution containing nutrients including lactic acid as a carbon and energy source was supplied into the medium to maintain the pH set-point of 7. Next, the effect of the lactic acid concentration in the feed solution on bacterial growth and physiology was evaluated. Finally, the effect of the concentration of sodium nitrate in the feed solution was evaluated. Taken together, a development pathway to intensified bioreactor cultures of *M. gryphiswaldense* MSR-1 is provided that could be scaled up in future work.

### A simple fermentation strategy to grow magnetosome-producing *M. gryphiswaldense* MSR-1

Preliminary experiments were used to determine the routine operational conditions of the pH-stat strategy. In these experiments, cellular magnetosome content was determined by measuring intracellular iron content using atomic absorption spectroscopy (AAS). In previous works, magnetosome production has been measured employing methods to determine dry weight of magnetosomes [14] using the protocol described by Xiang et al. [30], or AAS of purified magnetosomes. Alternatively, magnetosome yields have been calculated by measuring iron in media and subsequent mass balances [31], or by means of inductively coupled plasma optical emission spectroscopy [15]. Therefore, such variation in analytical methodologies to determine magnetosome production must be considered when comparing studies.

First, the effect of air supply to the culture (0 – 100 mL·min^−1^) and different stirring rates in the bioreactor (100–500 rpm) were evaluated. Setting air flow rates above 10 mL·min^−1^ and stirring above 300 rpm at the beginning of the fermentation prevented the pO_2_ from dropping to 0%, and thus the low oxygen tension required for magnetosome formation could not be achieved. The position of the two impellers in the bioreactor was optimized to minimize foaming; the upper impeller was placed 2 cm below the liquid/air interface with the lower impeller placed 3 cm below the upper impeller. Together with the low aeration rates, this suppressed foaming and therefore prevented the requirement for antifoam, which can inhibit growth [13].

Supply of oxygen and iron was investigated in two sets of two-stage fermentations, with the aim of temporally separating biomass and magnetosome formation, as is common practice in fermentations generating recombinant proteins, where growth and protein production are temporally separated [32]. First, *M. gryphiswaldense* MSR-1 was grown aerobically (150–400 rpm stirring, 10 mL·min^−1^ airflow, control of pO_2_ to >10% by varying stirrer speed, pH-stat feeding mode) in FSM medium containing a total iron concentration of 7.7 mg·L^−1^ (5.6 mg·L^−1^ supplied as ferric citrate (C_6_H_5_FeO_7_) and 2.1 mg·L^−1^ supplied as FeSO_4_ in the EDTA trace element solution) to an OD_565_ of around 6, which took 60 h. The bioreactor was then purged continuously with nitrogen gas (0.4 – 0.6 L·min^−1^) in order to rapidly decrease the pO_2_ to ‘zero’, aiming to reach conditions suitable for magnetosome production. This switch from aerobic to anaerobic conditions increased the iron content of the cells (determined using AAS) from 2 to 8.7 mg of iron per gram DCW, but cell growth ceased under anaerobic conditions. Previous studies carried out by Heyen and Schüler [2] growing MSR-1 under aerobic conditions in medium containing 100 μM ferric citrate determined basal levels of intracellular iron up to 10.8 mg·g^−1^ of DCW; these cells were classed as only weakly magnetic, probably due to oxygen limitation caused by high biomass concentrations. Therefore, our basal cellular iron contents are comparable to previous reports [2], indicating either formation of small quantities of magnetosomes, or more likely, non-magnetosome intracellular iron that could be stored by bacterioferritin under aerobic conditions [23].

Next, MSR-1 was grown under oxygen-limited conditions (pO_2_ ≤ 0.4%) using pH-stat mode for 54 h to an OD_565_ of around 4.3 in FSM without the addition of ferric citrate (this medium contained 2.1 mg of Fe^2+^·L^−1^, supplied as EDTA-TES). Then, a pulse of ferric chloride was added to the feed solution to bring the final concentration of Fe^3+^ to 414 mg·L^−1^; the feed also contained 1.4 mg of Fe^2+^·L^−1^, supplied as Mineral Elixir). This caused a dramatic cessation of cell growth after 3 h; the final biomass concentration achieved was OD_565_ ∼6.4, and cellular iron concentration increased from 3.1 to 6.1 mg·g^−1^ DCW after a total fermentation time of 71 h. These two scoping studies demonstrated that rapid changes in oxygen concentration or iron concentration were detrimental to overall process performance, thereby directing design of culture methods without such changes.

Following these initial scoping experiments, routine fermentation conditions were established as follows. Stirring rate was maintained at 250 rpm throughout. Bioreactor experiments were started without any air supply so that the drop in pO_2_ occurred within *ca.* 24 h of fermentation and reached ‘zero’ at an OD_565_ of 1 – 1.5. Air supply was started ∼2 h after reaching a pO_2_ of 0% at a flow rate of 3 mL·min^−1^; airflow rate was subsequently increased by 1 mL·min^−1^ for every 1 unit increase in OD_565_. This aeration strategy allowed cultures to start growing aerobically, then gradually adapt to the decreasing pO_2_, finally resulting in oxygen-limited conditions suitable for magnetosome formation. All the other fermentation conditions are described in the Materials and Methods section. Fig. S1 shows an example of changes in OD_565_, stirrer speed, pH, pO_2_ and airflow over a representative pH-stat fermentation.

Our growth strategy employs a simple set up approach, minimizes the use of complex control parameters, and eliminates the need for gas blending. This method permitted biomass concentrations comparable to previously reported work to be attained [15], and to the best of our knowledge only one academic research group [14,15] has recorded significantly higher MTB biomass yields, i.e. OD_565_ values of 30.4 and 43 in 7.5 L and 42 L bioreactors, respectively.

### Optimization of lactic acid supply in the feed solution

The use of lactate or lactic acid as a carbon source has been widely studied in *M. gryphiswaldense* bioreactor experiments. Heyen and Schüler [2] used flask standard medium (FSM) and large scale medium (LSM) containing 27 mM potassium lactate as a carbon source in batch experiments. 15 mM sodium lactate was used in optimized fermentation medium (OFM) by Sun and co-workers [31] and Li et al. [17] in batch and fed-batch experiments, respectively. Zhang and co-workers [14] sought to reduce accumulation of Na^+^ and Cl^−^ ions in the media, which were thought to inhibit growth. They used sodium lactate in the batch medium and three formulations of feed solution for pH-stat experiments with different concentrations of lactic acid or sodium lactate as carbon source, and NH_4_Cl or ammonium hydroxide as nitrogen source. They showed that the osmotic potential was maintained throughout the fermentation (100 mmol·kg^-1^) using Feed C, which contained 100 g·L^-1^ lactic acid and 18 mL·L^-1^ ammonium hydroxide, allowing achievement of the highest biomass concentration of *M. gryphiswaldense* MSR-1 to date (equivalent to OD_565_ = 30.4 in a 7.5 L bioreactor).

The Feed C of Zhang et al. [14] was used as a starting point to assess the effect of lactic acid concentration in the feed. Three oxygen-limited fermentations were performed with 50, 100 or 200 g·L^−1^ lactic acid in the feed. The pH of the feeds were 3.65, 3.05 and 2.65, respectively. As a consequence, the most acidic feed required less volume to maintain the pH set point (7.0 ± 0.05) during pH-stat growth. An aerobic fermentation was also carried out with 100 g·L^−1^ lactic acid feed. Fig. S2 shows the relationship between the quantity of feed solution added to bioreactors in pH-stat mode and the biomass concentration in g DCW·L^-1^; a linear regression was fitted to data where cells were actively growing, with R^2^ values of 0.94–0.99. The calculated feeding rates were respectively 131.4, 17.8 and 1.63 mL of feed per g DCW·L^-1^ for the 50, 100 and 200 g·L^-1^ lactic acid oxygen-limited experiments. The aerobic experiment resulted in a feeding rate of 2.92 mL of feed for every g DCW per litre. The lactic acid concentration decreased over the oxygen-limited fermentations employing 100 and 200 g·L^-1^ lactic acid, whereas it rose during the 50 g·L^-1^ lactic acid feed fermentation. This observation is relevant when considering the required volume of feeding solution, that in turn affects the production costs in an industrial setting. Significantly more 100 g·L^-1^ lactic acid feed was required for oxygen-limited growth than needed for aerobic growth. It should be noted that feed requirements are a function of pH increases in the fermenter and do not correlate directly to lactic acid demand as a carbon source; this reinforces the need to optimize lactic acid concentration in the feed, to balance pH maintenance, minimize cost and prevent accumulation of excess carbon source or other nutrients.

**Fig. 1:**
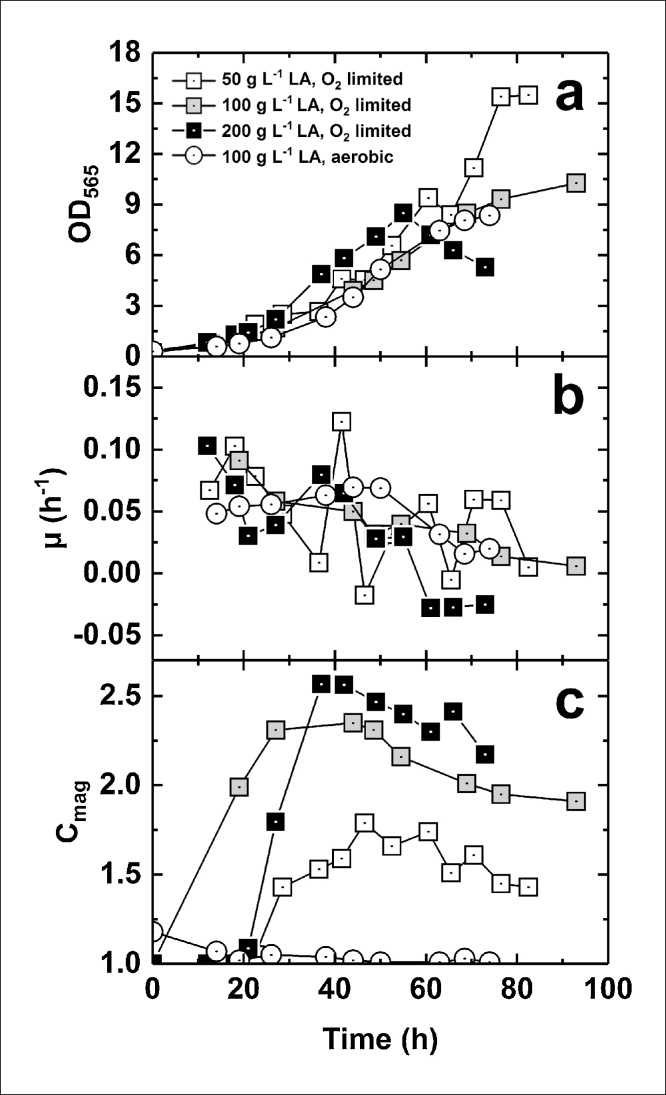
Comparison of oxygen-limited and aerobic fermentations conducted with different feed lactic acid concentrations. Plots show (a) OD_565_, (b) specific growth rate, μ, and (c) cellular magnetic response, C_mag_ versus time.

Growth curves (Fig. 1a) show that OD_595_ was highest in the culture grown with 50 g·L^−1^ lactic acid feed. Growth rates were calculated (Table 1) and it was observed that maximum growth rate (μ_max_) occurred at the early stage of the fermentation and within the first 24 h of culture in all microaerobic experiments, whereas growth rate remained roughly constant over the exponential phase in the aerobic experiment (Fig. 1b).

**Table 1:** Comparison of bioprocess parameters for cultures with varying feed lactic acid concentration.

Lactic acid concentration in feed (g·L^−1^)	μ_max_ (h^−1^)	μ^b^ (h^−1^)	μ^a^ (h^−1^)	Y_x/s_^b^	Y_x/s_^a^
50	0.12	0.056	0.051	0.15	0.26
100	0.09	0.069	0.02	0.23	0.23
200	0.1	0.07	−0.003	0.23	0.02
100 (Aerobic)	0.07	0.058*		0.14*	

Key: μ^b^ and μ^a^ correspond to the growth rate before and after reaching maximum C_mag_, and Y_x/s_^b^ and Y_x/s_^a^ are the corresponding yield coefficients; *values correspond to the exponential phase.

Cellular magnetism (C_mag_) was also measured to determine the response to cells to magnetic fields. Growth rates (Fig. 1b, Table 1) were generally higher for the period before (μ_b_) than the period after (μ_a_) the point at which the highest C_mag_ value was reached in oxygen-limited cultures (Fig. 1c). When comparing the aerobic and oxygen-limited fermentations carried out with the same feed composition, the latter showed a higher μ_max_ value followed by a decrease of μ throughout the exponential phase, as opposed to lower, but roughly constant μ in the aerobic experiment (Fig. 1b, Table 1). Our results showed lower μ values than reported previously for use of pH-stat mode; this may reflect differences in growth and operational strategies to maintain oxygen-limited conditions and/or media composition.

Our total fermentation time varied between 70–94 h as opposed to 44 h reported by Zhang and co-workers [14] to reach an OD_565_ of 30.4; our maximum OD_565_ value was 15.5. However, growth rates from our work (0.09 – 0.12 h^−1^) are only slightly lower than μ values obtained from oxystat experiments performed in previous studies with *M. gryphiswaldense*, which were ≈0.13 h^−1^ under microaerobic conditions, where OD_565_ values of <1.5 were reached [2].

Biomass to substrate yields for cultures were calculated both before (Y_x/s_^b^) and after (Y_x/s_^a^) reaching their maximum C_mag_ values (Table 1). Comparison of Y_x/s_^b^ within the oxygen-limited cultures revealed that the 50 g·L^−1^ lactic acid feed yielded less biomass per mass of supplied lactic acid than the 100 or 200 g·L^−1^ feeds. Y_x/s_^a^ values reflect the fact that the biomass increased after the oxygen-limited 50 and 100 g·L^−1^ fermentations reached maximum C_mag_. The 200 g·L^−1^ experiment did not increase in biomass after reaching maximum C_mag_.

**Fig. 2:**
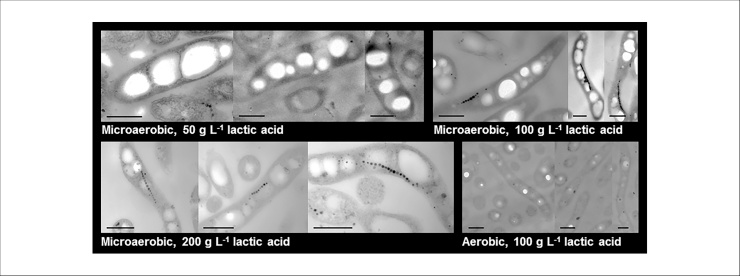
Transmission electron micrographs of bacteria from oxygen-limited and aerobic fermentations conducted with different feed lactic acid concentrations. The length of each scale bar is 0.5 μm.

Maximum biomass and magnetosome productivity for each fermentation are compared in the oxygen-limited experiments in Table 2. The amount of lactic acid in the feed solution inversely correlated to final biomass. The highest volumetric and specific magnetosome production, as determined by AAS, was achieved in the 200 g·L^−1^ feed experiment, yielding 56.8 mg iron·L^−1^ and 33.1 mg iron·g^−1^ DCW, respectively. The highest C_mag_ (2.56) was observed in the 200 g·L^−1^ feed experiment and the lowest with 50 g·L^−1^ feed, indicating that cellular iron content and C_mag_ do not correlate with one another. TEM images (Fig. 2a–d) showed that cells harvested from the 50 g·L^−1^ feed culture did not contain magnetosomes, or had short magnetosome chains, whereas longer chains were found in bacteria from the 100 g·L^−1^ and 200 g·L^−1^ feed fermentations. Additionally, as C_mag_ depends on changes in light scattering (as detailed in the Materials and Methods section), other parameters aside from the content of magnetosome chains, e.g. cell shape/morphology, likely influence the C_mag_ value. This possibility is explored below.

**Table 2:** Maximum biomass (OD_565_), iron concentration and C_mag_ measurements for fermentations presented in Fig. 1.

Maximum value	Lactic acid concentration (g·L^−1^)		
	50	100	200
OD_565_	15.5	10.3	8.5
Iron concentration (mg L^−1^)	53.5 ± 1.2	19.4 ± 0.2	56.8 ± 0.3
Iron/DCW (mg g^−1^)	14.8 ± 0.7	12.2 ± 0.3	33.1 ± 1.0
C_mag_	1.79	2.35	2.56

For iron concentration values, mean values from 3 measurements are stated ± standard deviation.

Maximum C_mag_ values were achieved at an OD_565_ of 5–8 depending on the experiment (Fig. 1c), but in all cases did not correspond to the point of maximum biomass concentration. This observation can be compared with previous studies where highest C_mag_ values were achieved at OD_565_ values of 2–5 in pH-stat cultures [15,23]. Our results demonstrate the importance of not only the chemical nature of the carbon source, which has been previously described by other research groups, but also its concentration within the feed. While the 50 g lactic acid L^−1^ feed culture resulted in the highest biomass achieved using our growth strategy, the highest cellular iron content and C_mag_ were obtained using the highest lactic acid feed concentration of 200 g·L^−1^. Therefore, a compromise between biomass and magnetosome production must be considered in industrial settings.

### Application of flow cytometry to monitor physiology

Flow cytometry (FCM) was recently used by our group as a rapid, single-cell technique to study the physiology of MTB during growth in flasks [24]. In the present study, we have applied FCM to analyze and optimize fermentations. Propidium Iodide (PI) is a red DNA dye widely used to detect dead bacteria; the intact membrane of viable bacteria excludes PI, therefore live cells are PI^−^ and dead cells are PI^+^. DiBAC_4_(3) (Bis-(1,3-Dibutylbarbituric Acid) Trimethine Oxonol), commonly referred to as Bis-oxonol (BOX), is a green lipophilic dye that enters the cell only if the membrane is depolarized [33] allowing determination of cellular respiration. Co-staining with PI and BOX therefore permits detection of three physiological states: ‘healthy’ (PI^−^ BOX^−^); ‘injured’ (PI^−^ BOX^+^; the cells are intact but are depolarized); and ‘dead’ (PI^+^ BOX^+^).

**Fig. 3:**
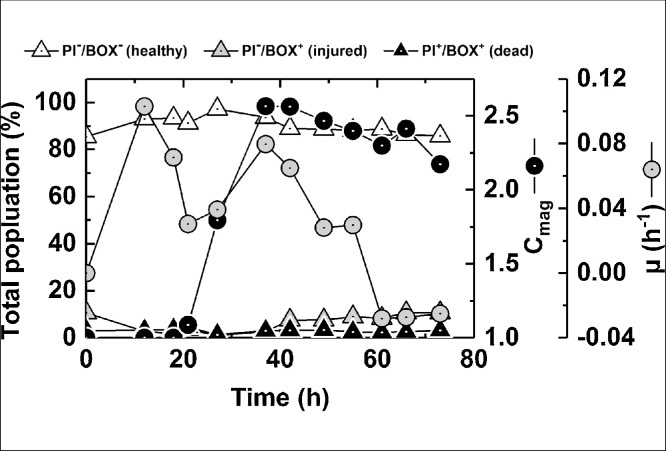
Assessment of bacterial physiology using FCM. Bacteria from an oxygen-limited pH-stat culture with 200 g·L^−1^ lactic acid and 6 g·L^−1^ sodium nitrate in the feed were stained with PI and BOX and analyzed by FCM. The percentages of cells within three differently stained populations, i.e. PI-/BOX- (‘healthy’), PI-/BOX^+^ (‘injured’) and PI^+^/BOX^+^ (‘dead’), are plotted.

Co-staining with PI and BOX showed similar results in experiments with different feed lactic acid concentrations (Fig. 3). It was observed that between 85–90% of cells were healthy at the start of fermentation. This relatively low proportion of healthy cells may reflect the use of a late stationary phase inoculum as is observed with *E. coli* (TW Overton, unpublished data). The size of the healthy population increased over time, reaching nearly 99% after ∼27 h, roughly coinciding with the increase in C_mag_ from 1 to 2.5 between 20 h and 35 h. Subsequently the healthy population dropped to 80 – 85% corresponding to the period (35–72 h), where growth slowed and C_mag_ fell to <2.25. The proportion of injured cells (PI^−^ BOX^+^) was highest when the healthy population was lowest, whereas the proportion of dead cells (PI^+^ BOX^+^) remained low (1–5%) throughout the fermentation.

### Application of flow cytometry to monitor cell size, shape and PHA accumulation

**Fig. 4:**
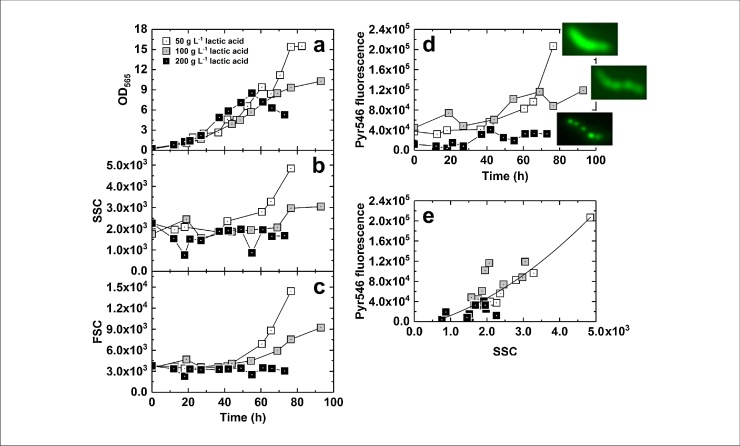
Flow cytometry analysis of scatter and PHA content of cells grown with different feed lactic acid (LA) concentrations. Samples taken from oxygen-limited pH-stat fermentations with different feed lactic acid concentrations were analyzed by measurement of OD_565_ and FCM. Twenty-five thousand data points were collected for each sample and mean values are represented. Panels show OD_565_ (a), forward scatter, FSC (b), side scatter, SSC (c), and fluorescence post-staining with Pyr546 (d) plotted against time; and Pyr546 fluorescence vs. SSC (e). The insets in (d) show fluorescence micrographs of cells at the end of each fermentation after staining with Pyr546.

Cell size and optical complexity were monitored by FCM by means of light scattering. Light scattered by cells is measured by two detectors: forward scatter (FSC), measured in line with the illuminating laser beam, broadly correlates with cell size; side scatter (SSC), measured orthogonally to the laser beam, indicates the granularity or optical complexity of cells [34]. Comparison of FSC and SSC data for cultures with different lactic acid feed concentrations revealed that the largest increases in both FSC and SSC were noted for cultures fed with lactic acid at 50 g·L^−1^ and the smallest were observed when a feed concentration 200 g·L^−1^ was employed (Fig. 4a–c). TEM micrographs confirm differences in cell size and granularity; cells fed with 50 g·L^−1^ of lactic acid (Fig. 2a) exhibited a rounder shape with large white inclusions, resembling PHA aggregates as reported by [35,36] whereas cells fed with 100 g·L^−1^ (Fig. 2b) and 200 g·L^−1^ lactic acid (Fig. 2c) were more elongated.

Given that TEM (Fig. 2) revealed that cells fed with different concentrations of lactic acid contained differing amounts of PHA granules, FCM was used to determine cellular PHA accumulation by staining of the cells with the green lipophilic dye Pyrromethene-546 (Pyr546; Fig. 4d) [37,38]. As with the FSC and SSC data (Fig. 4b-c), Pyr546 fluorescence (and thus PHA concentration per cell) was highest for cells fed with 50 g·L^−1^ lactic acid and lowest for 200 g·L^−1^ lactic acid feed. The correlation between SSC and Pyr546 fluorescence is plotted in Fig. 4e. Fig. 4d also shows fluorescence micrographs of cells taken from the end of each fermentation stained with Pyr546, corroborating FCM and TEM data. The increased quantity of PHA in cells grown with 50 g·L^−1^ lactic acid would also explain why the Y_x/s_ values for this culture were lower than those recorded with the higher lactic acid feed concentrations (Table 1). Histograms of Pyr546 fluorescence (shown in Fig. S3) demonstrate, that for cultures fed with 50 and 100 g·L^−1^ lactic acid, a narrow normal distribution was found, meaning that all cells within the culture exhibited similar levels of PHA accumulation. In contrast, cells fed with 200 g·L^−1^ lactic acid displayed bimodal distribution at two time-points (19–22 h and 74–96 h), indicating two populations within the culture, one PHA-rich, the other comparatively PHA-poor.

The formation of PHA aggregates in MTB has been previously reported. PHA is generated when there is an excess of carbon source compared to another nutrient, frequently nitrogen. As the 50 g·L^−1^ lactic acid feed experiment had the highest quantity of carbon source added to the bioreactor, and lactic acid was seen to accumulate in the medium, it is logical that highest PHA accumulation occurred under these conditions. In addition, excess reducing power in MTB is consumed through PHA formation and hydrogen release [39]; this phenomenon has been observed in several studies under different culture conditions [40]. In this work, we demonstrate for the first time the formation of PHA in *M. gryphiswaldense* high-cell density bioreactor cultures. Our results are in agreement with previously reported studies regarding the energy competition between PHA and magnetosome formation [41], the 200 g·L^−1^ lactic acid feed experiment resulting in the highest magnetosome production (33.1 mg iron·g^−1^ DCW) and lowest PHA formation.

### Nitrate enhances cell growth

Previous studies reported that magnetosome formation and denitrification occur simultaneously under oxygen-limited conditions [19]. The nitrate concentration has been optimized for media used in shake-flask experiments, but to the best of our knowledge no research has focused on the optimal supply of nitrate in relatively high cell density *M. gryphiswaldense* bioreactor cultures. Hence, we optimized the supply of sodium nitrate into the feed solution using 100 g·L^−1^ lactic acid as a carbon source in pH-stat cultures.

**Fig. 5:**
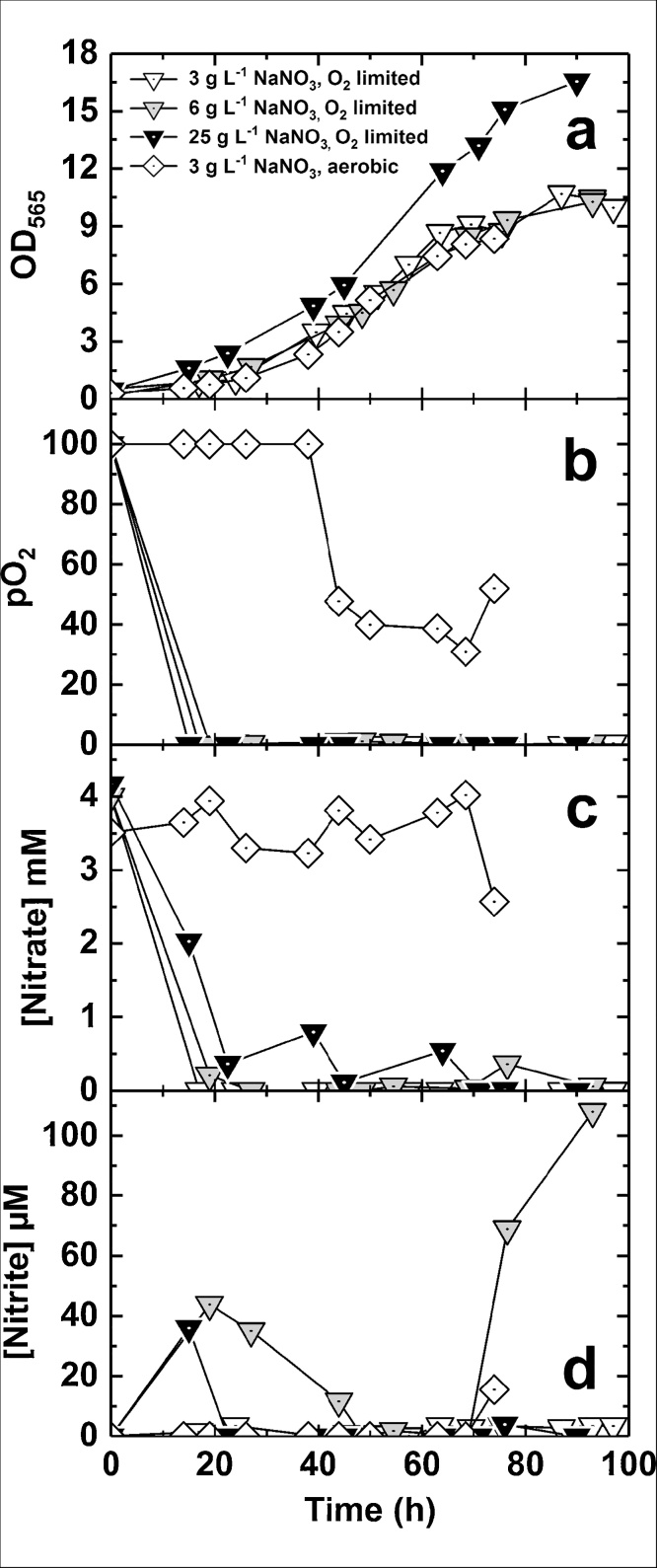
Comparison of oxygen-limited and aerobic fermentations conducted with different feed nitrate concentrations. Panels show OD_565_ (a), pO_2_ (b), and nitrate (c) and nitrite (d) concentrations plotted against time. Samples were measured in duplicate for nitrate and nitrite analysis and in triplicate for analysis of iron content. Mean values are plotted.

Fig. 5a–d show plots of OD_565_, pO_2_, medium nitrate and nitrite concentrations versus time in culture for oxygen-limited experiments conducted with feed solutions containing 3, 6 or 25 g·L^−1^ NaNO_3_. The lactic acid concentration in the feed was 100 g·L^−1^ for each experiment. For comparison, an experiment was also performed under aerobic conditions (pO_2_ > 30%) with 6 g·L^-1^ NaNO_3_ in the feed. The nitrate concentration did not vary significantly during the aerobic experiment, remaining around 4 mM during the exponential phase and decreasing to 2.6 mM at the end of the fermentation (Fig. 5c). The concentration of NO_3_- initially present in FSM was 4 mM (0.34 g·L^-1^) and, despite 54 mmol of NO_3_- being added to the fermenter during feeding (768 mL of feed was added), the NO_3_- concentration was steady (Fig. 5d). Nitrite concentration was also very low throughout, except for the last sampling point (15.6 μM NO_2_-). This suggests concurrent denitrification and aerobic respiration; this has previously been observed by Li et al. [24]. For oxygen-limited growth, nitrate was rapidly utilized when the pO_2_ decreased to zero (Fig. 5b), and remained at very low concentrations in all experiments (Fig. 5c). In each case, nitrite concentrations transiently increased (Fig. 5d) following the onset of nitrate utilization (Fig. 5c), then decreased; nitrite concentration rose slightly during the course of some fermentations (Fig. 5d).

The effect of nitrate concentration in the feed had an impact on several bioprocess parameters (Table 3). Maximum growth rate (μ_max_) was comparable for all cultures, whereas higher biomass concentration was achieved in the culture fed with 25 g·L^−1^ sodium nitrate. The maximum growth rate was observed within the first few hours of oxygen-limited cultures. Growth rates before the point at which maximum C_mag_ was achieved (μ_b_) did not show a clear correlation with nitrate concentration; however, all cultures had significantly decreased growth rates after the peak C_mag_. Biomass to substrate yields (Y_x/s_) before the time of maximum C_mag_ showed similar values except for the experiment with 3 g·L^−1^ NaNO_3_. FCM analysis (Fig. S4) shows that cells from cultures with the lowest nitrate feed concentration accumulated the most PHA, and had higher FSC and SSC (indicating a change in morphology) than cells grown at higher nitrate concentrations.

**Table 3:** Comparison of bioprocess parameters for cultures with varying feed sodium nitrate concentration.

NaNO_3_ concentration in feed (g·L^−1^)	μ_max_ (h^−1^)	μ^b^ (h^−1^)	μ^a^ (h^−1^)	Y_x/s_^b^	Y_x/s_^a^
3	0.08	0.047	0.018	0.55	0.3
6	0.09	0.069	0.02	0.23	0.23
25	0.08	0.052	0.015	0.26	0.23
6 (Aerobic)	0.07	0.058*		0.14*	

Key: μ^b^ and μ^a^ correspond to the growth rate before and after reaching maximum C_mag_, and Y_x/s_^b^ and Y_x/s_^a^ are the corresponding yield coefficients; *values correspond to the exponential phase.

Correlation of biomass and magnetosome production with nitrate supply was also investigated as shown in Table 4. The highest concentration of nitrate in the feed resulted in highest biomass concentration and quantity of cellular iron per litre of culture. However, C_mag_ values were >2.1 for all 3 nitrate concentrations, indicating highly magnetic cultures. Overall, whereas high feed nitrate concentration did not significantly increase the C_mag_ of bacteria, it did generate higher biomass concentration, and thus more magnetosomes per unit volume. Our results indicate that if pH-stat mode is used, NaNO_3_ should be included in the feed solution to allow enhancement of magnetosome and biomass production. Future work could determine if higher nitrate concentrations are able to support higher biomass concentrations.

**Table 4:** Peak biomass (OD_565_), iron concentration and C_mag_ measurements for pH stat cultures presented in Fig. 5.

Maximum value	NaNO_3_ concentration (g·L^−1^)		
	3	6	25
OD_565_	10.7	10.3	16.6
Iron concentration (mg L^−1^)	19.2 ± 0.2	19.4 ± 0.3	54.3 ± 0.4
Iron/DCW (mg g^−1^)	14.1 ± 0.3	12.2 ± 1.0	16.4 ± 0.35
C_mag_	2.58	2.35	2.14

For iron concentration values, mean values from 3 measurements are stated ± standard deviation.

## Conclusions

We have developed a simple strategy for production of *M. gryphiswaldense* MSR-1 by employing a pH-stat operational mode adapted from previous studies. The advantage of this strategy is that it does not require tight and sophisticated control tools (gas blending, extremely sensitive oxygen probes) to achieve efficient biomass and magnetosome production. Biomass concentrations were obtained comparable to the highest published values to date [14,15] using comparable analytical techniques. The concentration of two key nutrients in the feed solution, lactic acid and nitrate was also investigated. Lower concentrations of lactic acid in the feed increased final biomass concentration, whereas a high concentration increased cellular magnetism. There is therefore a need to balance the production of biomass and magnetosomes in the design and operation of these fermentation processes. The highest nitrate concentration tested (25 g·L^−1^) gave rise to the highest biomass concentration.

Flow cytometry has been shown to be a useful analytical strategy for the determination of bacterial physiology, morphology and PHA content, which can be used to guide process development. Bacterial ‘health’ was steady throughout fermentations. Cellular PHA content was shown to be inversely correlated to feed lactic acid concentration, and also correlated with scatter measurements.

Our study represents significant progress towards the implementation of rapid analytical techniques that will aid in the manufacture magnetosomes in industrial settings, itself a prerequisite of the application of magnetosomes in clinical and biotechnological applications. In conclusion, our work provides the research community with a relatively simple method to produce large amounts of magnetosomes using in *M. gryphiswaldense* MSR-1 grown in bioreactors.
