# Supplementary material for: Development of a simple intensified fermentation strategy for growth of Magnetospirillum gryphiswaldense MSR-1: Physiological responses to changing environmental conditions
Source: N Biotechnol. 2018 Nov 25;46:22–30. doi: 10.1016/j.nbt.2018.05.1201 (PMC6109776; doi:10.1016/j.nbt.2018.05.1201)
Supplement: Supplementary file 1 [file mmc1.pdf]

Supplemental information for:

Development of a simple intensified fermentation strategy for growth of *Magnetospirillum gryphiswaldense* MSR-1: physiological responses to changing environmental conditions

Alfred Fernández-Castané[1,2*], Hong Li[1], Owen RT Thomas[1], Tim W Overton[1,2,§]

[1]School of Chemical Engineering and [2] Institute for Microbiology & Infection, University of Birmingham, B15 2TT Birmingham, UK.

*Current address: Aston Institute of Materials Research & European Bioenergy Research Institute, Aston University, Birmingham, B4 7ET, UK.

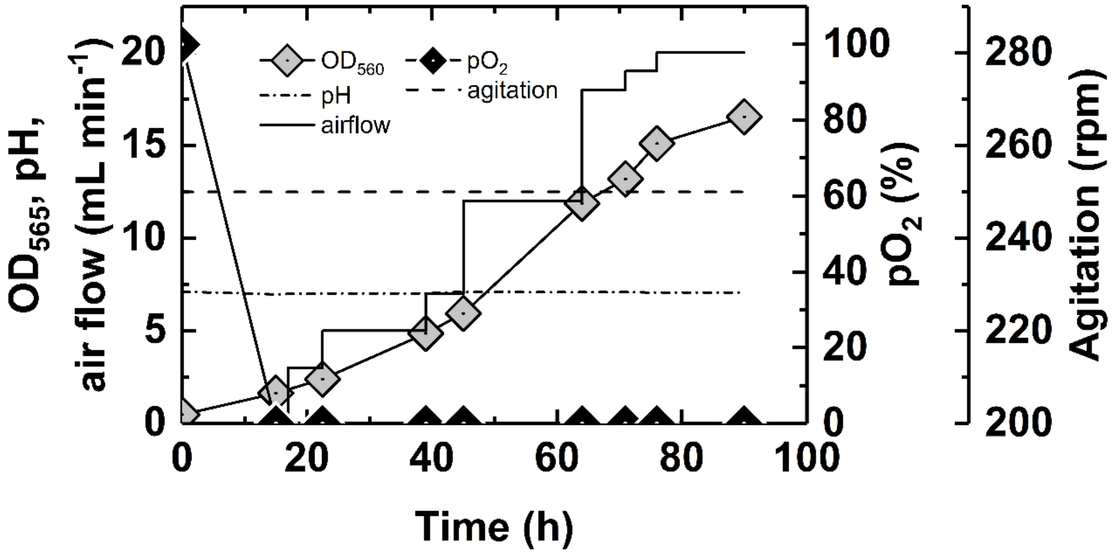

Figure S1. A representative pH-stat fermentation. MSR-1 was grown in FSM medium with a feed comprising 100 g·L^{-1} lactic acid and 25 g·L^{-1} sodium nitrate. Other parameters as explained in Results and Discussion and Materials and Methods sections.

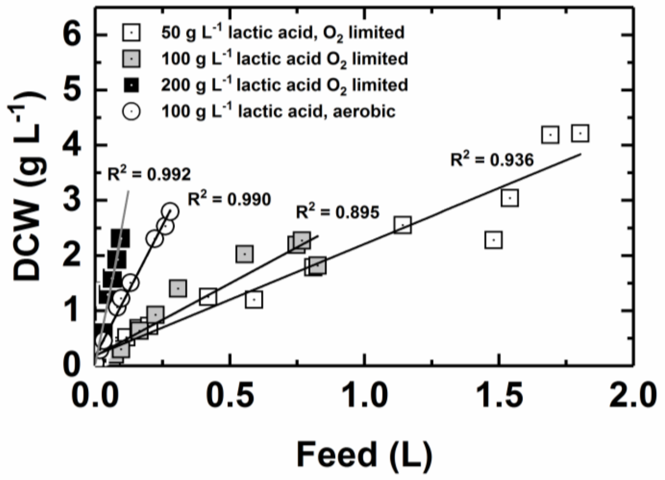

Figure S2. Correlation between feed volume supplied and biomass concentration. Oxygen-limited cultures with feed lactic acid concentrations of 50 g·L^{-1} (white squares), 100 g·L^{-1} (gray squares), and 200 g·L^{-1} (black squares); aerobic culture with feed lactic acid concentration of 100 g·L^{-1} (white circles).

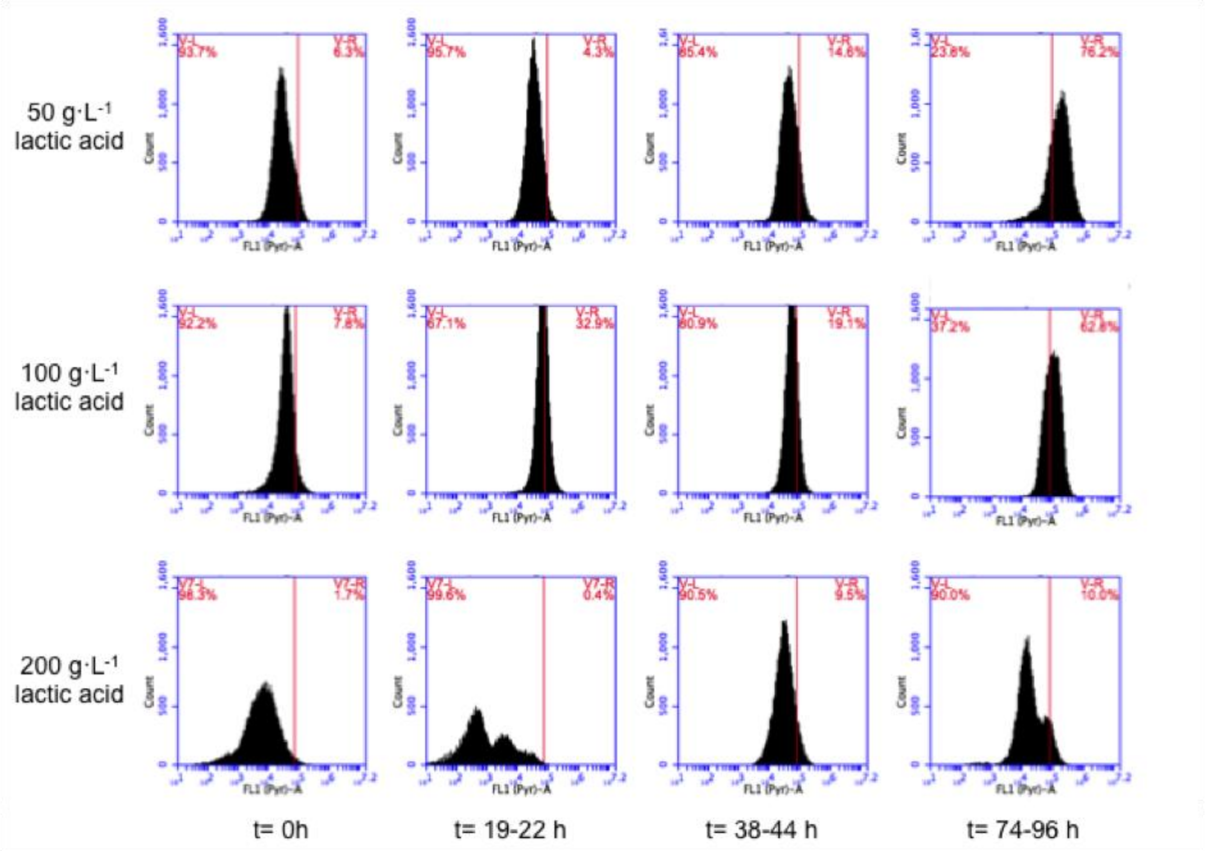

Figure S3. Fluorescence intensity histograms of samples collected at 0h (start of fermentations), 19–22 h, 38–44 h or 74–96 h (end of fermentation) of oxygen-limited cultures containing 50, 100 or 200 g·L^{-1} lactic acid in the feeding solution.

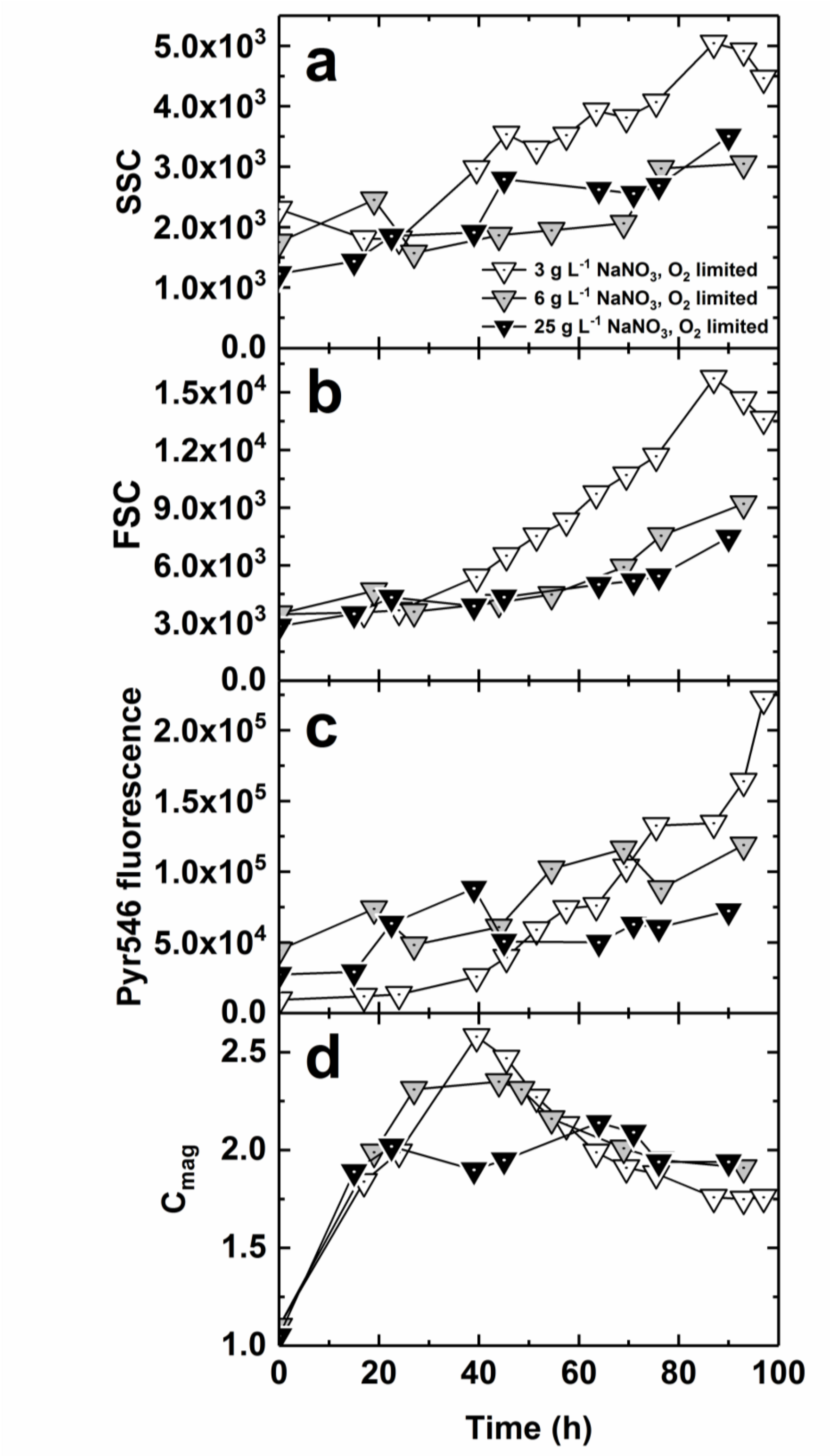

Figure S4. FCM and C_{mag} analysis of cells grown with different feed sodium nitrate concentrations. Plots show side scatter, SSC (a), forward scatter, FSC (b); fluorescence of Pyr546-stained cells (c), and C_{mag} values (d) plotted against time.

For FCM 25,000 data points were collected for each sample and mean values are represented. Symbols: oxygen-limited pH-stat cultures with feed $NaNO_3$ concentrations of 3 $g{\cdot}L^{-1}$ (white down-triangles), 6 $g{\cdot}L^{-1}$ (gray down-triangles) and 25 $g{\cdot}L^{-1}$ (black down-triangles).

Supplemental Table S1. List of fluorescent dyes used for fluorescence assays

Dye name	Abbreviation	Excitation maximum (nm)	Emission maximum (nm)	Stock concentration	Solvent	Working concentration	Fluorescence channel and filters
$DiBAC_4(3)$ (Bis-(1,3-Dibutylbarbituric Acid) Trimethine Oxonol,	BOX	490	516	10 mg·mL^{-1}	DMSO	100 ng/mL	FL1 (533/30 BP Green)
Propidium iodide	PI	533	617	200 µg·mL^{-1}	dH_2O	100 ng/mL	FL3 (670 LP Red)
Pyrromethene-546	Pyr546	493	519	0.1 mg·mL^{-1}	10 % DMSO	0.5 µg/mL	FL1 (533/30 BP Green)
PhenGreen SK	PG-SK	525	580	1 mM	DMSO	5 µM	FL1 (533/30 BP Green)
